# Capillariaisis (Trichurida, Trichinellidae, *Capillaria hepatica*) in the Brazilian Amazon: low pathogenicity, low infectivity and a novel mode of transmission

**DOI:** 10.1186/1756-3305-3-11

**Published:** 2010-02-26

**Authors:** Luis Marcelo Aranha Camargo, Juliana de Souza Almeida Aranha Camargo, Luana Janaina de Souza Vera, Pedro di Tarique Crispim Barreto, Eudes Kang Tourinho, Marcia Maria de Souza

**Affiliations:** 1 Department of Parasitology, University of Sao Paulo, Sao Paulo, Brazil; 2 Department of Medicine, São Lucas Faculty, Rondonia, Brazil; 3 University of Rondonia, Rondonia, Brazil; 4 9 de Julho Hospital, Rondonia, Brazil; 5 Oswaldo Cruz Foundation, Bahia, Brazil

## Abstract

**Background:**

Human capillariasis caused by *Capillaria hepatica (syn. Calodium hepaticum) *is a rare disease with no more than 40 cases registered around the world. Classically, the disease has severe symptoms that mimic acute hepatitis. Natural reservoirs of *C. hepatica *are urban rodents (*Mus musculus *and *Rattus novergicus*) that harbor their eggs in the liver. After examining the feces of 6 riverine inhabitants (Rio Preto area, 8° 03'S and 62° 53' W to 8° 14'S and 62° 52'W) of the State of Rondonia, Brazil, and identifying *C. hepatica *eggs in their feces, the authors decided to investigate the real dimension of these findings by looking for two positive signals.

**Methods:**

Between June 1^st ^and 15^th^, 2008, 246 out of 304 individuals were clinically examined. Blood samples were collected, kept under -20°C, and test by the indirect immunofluorescence technique.

**Results:**

The first positive signal was the presence of specific antibodies at 1:150 dilution, which indicates that the person is likely to have been exposed to eggs, most likely non-infective eggs, passing through the food chain or via contaminated food (total prevalence of 34.1%). A second more specific signal was the presence of antibodies at higher titers, thus indicating true infection.

**Conclusions:**

The authors concluded that only two subjects were really infected (prevalence of 0.81%); the rest was false-positives that were sensitized after consuming non-embryonated eggs. The present study is the first one carried out in a native Amazonian population and indicates the presence of antibodies against *C. hepatica *in this population. The results further suggest that the transmission of the parasite occurs by the ingestion of embryonated eggs from human feces and/or carcasses of wild animals. The authors propose a novel mode of transmission, describing the disease as a low pathogenic one, and showing low infectivity.

## Background

Capillariasis is a cosmopolitan helminthiasis with zoonotic characteristics. Its etiological agent infects many species of mammals, birds, fish and invertebrate animals [1-10]. The etiological agents belong to the phylum Nematoda, order Trichurida, family Trichinellidae and genus *Capillaria*. This genus contains 300 species of which three are well known human parasites, namely: *Capillaria philippinensis, C. aerophila *and *C. hepatica*, which infect the bowels, the lower breathing airways and the liver, respectively [11].

Traditionally, most authors consider liver capillariasis a rare disease. Among at least 40 cases of parasitism caused by *C. hepatica *that have been registered in the literature, five occurred in Brazil [12,13]. Moreover, eggs of *C. hepatica *have been identified in the feces of individuals of Brazilian indigenous populations [14,15].

Eggs can be ingested from the soil or from carcasses of dead animals. Animal models have shown that the larvae of *C. hepatica *hatch at the level of the cecum, penetrate the mucous membrane and reach the porta system until they lodge in the hepatic parenchyma. Finally, after 1 month, they become adult worms. Fertilized eggs spread in groups around the females, which die within thirty days. The eggs remain viable and immature for approximately 120 days. In order for embryogenesis to occur, the eggs must reach the environment, which in this case is possible only after the death of the infected host and disintegration of its carcass. Alternatively, ingestion of organs (eg., liver) containing non-embryonated eggs and their further elimination through the feces, will also induce embryogenesis, thus resulting in infective eggs in the soil [16,17].

Clinical manifestations are related to the inflammatory reaction it causes in the adjacent tissues. Because they affect the hepatic tissue, clinical forms similar to those of acute viral hepatitis and the classical triad (fever, hepatomegaly and eosinophilia) may be present [18]. After diagnosing 6 riverine inhabitants of the State of Rondonia, Brazil, eliminating eggs of *C. hepatica *in their feces, the authors decided to investigate the real dimension of these findings by looking for positive signals at two levels. The first positive signal was the presence of specific antibodies at the 1/150 dilution, which indicates that the person is likely to have been exposed to eggs, most likely non-infective eggs, passing through the food chain or via contaminated food. A second more specific signal was the presence of antibodies at higher titers, thus indicating true infection.

## Methods

### Study Area

This work was performed in the locality of the Preto River, at the intersection of Machado River with Madeira River, Rondonia State, western Brazilian Amazon.

**Figure 1:**
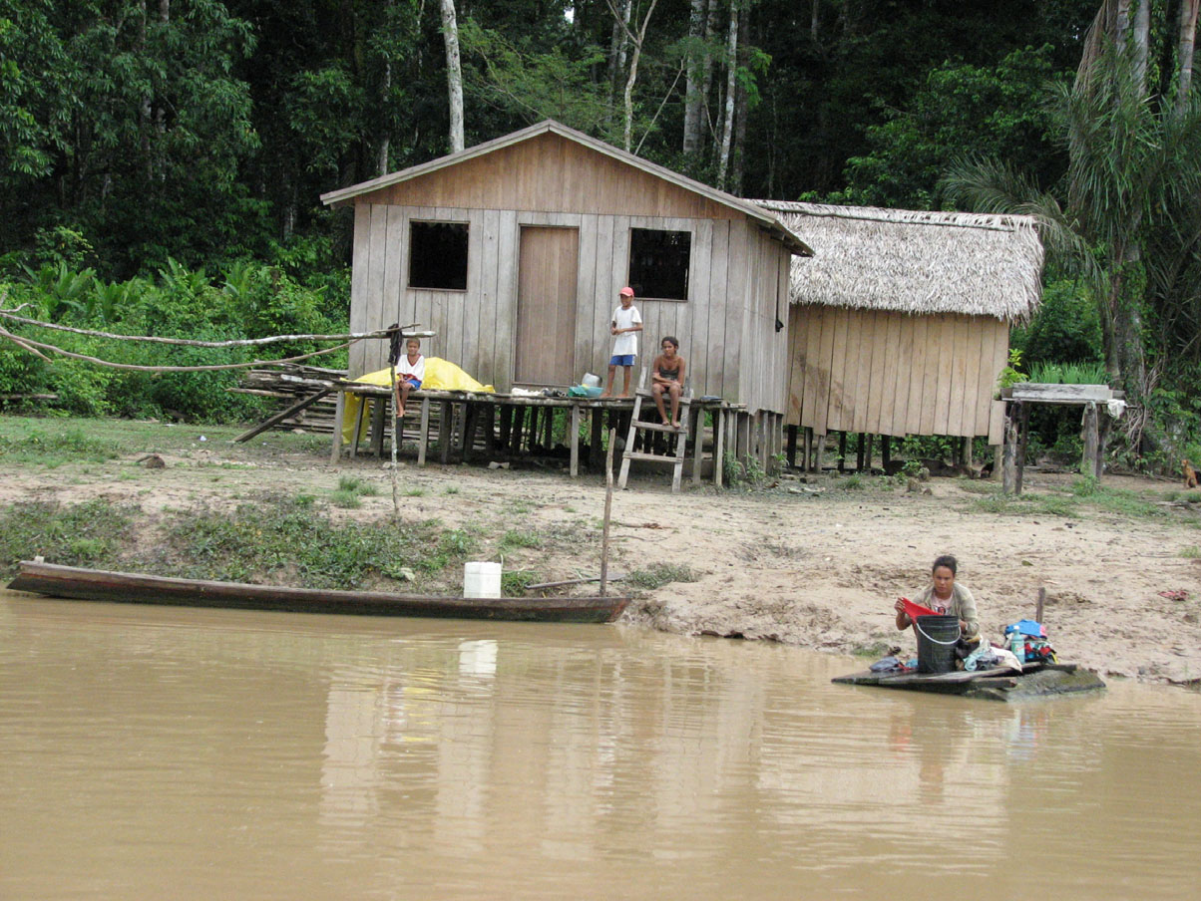
**Typical house and riverine inhabitants along the Rio Preto river, Rondonia, Amazon region, Brazil**.

The study area (8° 03'S and 62° 53' W to 8° 14'S and 62° 52'W) is inhabited by 304 individuals. These people are descendants of the "rubber soldiers" of the XIX and XX centuries, of native populations, and from immigrants from the south and southwestern regions of the country. This area lacks electricity and medical care. Houses are made of wood with thatched or asbestos roofs (Fig. 1). There are no toilets and excrements are thrown around the houses or dumped in cesspits located 10 to 20 meters from the houses. Water is taken from the river or from small wells known as "cacimbas" and it is consumed unfiltered and without any treatment. People earn their living working the land for their subsistence, selling the excess product to bargaining small merchants. Some people sell the pulp of native fruits (cacao, cupuassu, etc.) in Porto Velho, located more than 250 km away. The basic diet is fish, manioc flour, seasonal fruits and wild animals, such as monkeys, peccaries (*Tayassu spp*.), paca (*Agouti paca*), cotia (*Dasyprocta aguti*), deer, alligators, birds, etc.

### Population Data Collection

In the past 6 years, researchers from the University of São Paulo have been studying the Preto River area, registering its population. During this period the researchers diagnosed 6 inhabitants who presented *C. hepatica *eggs in their feces. Because the community is very small, all individuals were examined by the medical staff, except those who were not present in the community between June 1^st ^and 15^th^, 2008. From the 304 permanent inhabitants, 246 were present at the time of the study and all were examined.

#### Collection and processing of biological samples

Subjects read and signed a term of consent prior to the collection of samples. A volume of 10 mL of blood was collected from each subject in dry tubes by venous puncture, after the due asepsis of the forearm. Blood samples were kept at room temperature for 3 to 4 hours and then centrifuged at 3,000 rpm for 10 minutes. Each serum sample was kept at -20°C, until the serological exam was carried out.

Serum samples were diluted in saline buffered solution (PBS) at 50% to 1/150 and tested against paraffin cuts containing mice livers with *C. hepatica *eggs and worms, according to the technique proposed previously [19]. The same author suggests a cut-off of 1/400 to diagnose false from- true-positive cases. False-positive cases occur after the ingestion and immunological sensibilization by non-embryonated eggs. On the other hand, true-positive cases are the ones that ingested embryonated eggs and harbor the worms in the liver tissue. Results proved to be positive (>1/150) were scored as weak (PI), median (PII) and strong (PIII), based on fluorescence intensity, according to personal criteria of two different examiners. Nine of the 13 serum samples with median or strong reactions were further diluted to 1:500 and 1:1000 (higher than the cut-off level) in PBS and re-tested, in an attempt to exclude cases with unspecific fluorescence as suggested by Assis [19] The same technique was tested by Junker-Voss [20], who demonstrated a high specificity and sensibility to *C. hepatica*, with no cross-reactivity for serum samples of patients with documented infections of *Echinococcus granulosus*, *E. multilocularis*, *Fasciola. hepatica*, *Schistosoma mansoni*, *Toxocara canis*, *Trichuris trichiura*, and *Trichinella spiralis*.

Complementary laboratory tests (haemogram, AST/ALT, Billirubin, Gamma-glutamyltransferase, platelets count, B and C hepatitis serology, cytomegalovirus serology, feces parasitological exams) and ultrasonographic examination of the liver aiming to find alterations that could characterize hepatic capillariasis were performed for all the serological positive patients at the same time and using the same biological samples.

### Statistical Analyses

Information was stored in an Excell^® ^data file and analyzed by Epi Info 6.2. Qualitative variables (sex, age groups, positive/negative test and place of origin) were analyzed using the chi-square test and multivariate logistic regression. Quantitative variables (time living in the Amazon and in the study area, family income and age) were analyzed through the nonparametric Mann-Whitney U test, since these variables showed a non-normal distribution for the Shapiro-Wilk test. The significance level was set at 0.05.

### Ethical approval

This study was submitted to the Ethics Committee of the São Lucas School, registered under number PT/CEP/10/08.

## Results

A total of 246 subjects (81%) out of the 304 permanent inhabitants of the community had their serum samples examined. The population was basically composed by 194 native individuals (79%) and 52 (21%) people currently living in the area but original from outside the Amazon (immigrants). 41% reported to earn their living from agriculture and fishing, 28% performed activities related to home care and 28% were students. The rest of the population was composed of civil servants and small dealers. When examined, 10 individuals (4%) presented positive haemoscopy for malaria (*Plasmodium vivax and P. falciparum*).

Over ninety percent (91.7%) of the people interviewed informed they had eaten paca and/or agouti, and/or peccary meat within the previous 15 days, suggesting that this is a frequent habit in this population.

In order to verify if the remaining inhabitants of the community (19%) that were not sampled in the present study had a different profile from the sampled group tested by serology, we performed a statistical analysis (qui-square test, multivariate logistic regression and Mann-Whitney U test) of certain variables (sex, age, place of origin, time of residence in Rondonia and in the study area) and no significant statistical differences was found, thus suggesting that the sampled population is representative of our findings.

Of the total number of subjects, 84 showed weak, median, or strong positive serology at the 1/150 dilution, representing a total prevalence of 34.1%. Subjects who showed weak positive serology were in greater proportion than patients in the other two categories (x^2 ^= 101.2, p < 0.0001).

There was no statistic significance between seropositivity among the three age groups: 0 to 15 years (children group), 16-60 years (young group), and >60 (elderly group) (x^2 ^Yates corrected = 0.48, 2 df., p = 0.7867). Additionally, no significance was detected among the two sexs (x^2 ^Yates corrected = 2.34, df = 2, p = 0.1277).

However, when the place of origin (Amazon or outside Amazon) was compared by the qui-square test, those original from the Amazon region presented significantly more soropositivity (79/194 inhabitants, 40.7%) when compared to inhabitants original from outside the Amazon (5/52 soropositive, 9.6%) (x^2 ^= 17,65, p < 0.0001).

After controlling the covariates, the only significant factor related to capillariasis was the individual's place of origin (from Amazon) (odds ratio 3.9219, CI 95%, 1.1089-13.8702). In other words, Amazonians have 4 times more chance of developing capillariasis then those born elsewhere. No significant association was found between capillariasis and age or sex. (Table 1).

**Table 1:** Multivariate logistic regression of factors related to the occurrence of capillariasis, Rondonia, Amazon region, Brazil, 2008.

Variables	Odds Ratio (IC 95%)	β_i_	Standard Error	P-Value
Amazon origin	3,9219 (1,1089 -- 13,8702)	1,3666	0,6445	0,0340
Sex	1,4609 (0,8526 -- 2,5030)	0,3790	0,2747	0,1677
Age Group (16-60)	1,3882 (0,7950 -- 2,4238)	0,3280	0,2844	0,2488
Age Group (> 60)	1,9175 (0,5728 -- 6,4193)	0,6510	0,6165	0,2910
Intercept	#	-0,1658	0,2560	0,5171

Of the 13 positive cases classified as median or strong, nine samples were re-examined at 1: 500 and 1:1000 dilutions. Maintenance of immunofluorescence at these dilutions is considered to be specific for *Capillaria hepatica *[19]. Of these, only 2 sera (one 4 years-old boy and one 34 years-old woman) remained positive after the dilution to 50% with PBS, thus characterizing a strong seroprevalence of only 0.81% among the sampled population. All nine patients were subjected to complementary laboratory tests (haemogram, AST/ALT, bilirrubin, gamma-glutamyltransferase, platelets count, B and C hepatitis serology, cytomegalovirus serology, and feces parasitological exams) and ultrasonographic examination of the liver aiming to find alterations that could characterize hepatic capillariasis. All results were normal for the nine patients.

## Discussion

The target population is young (averaging 26 years) and has been living in Rondonia for 21 years, 14.8 of those years in the Rio Preto area. Most of the population is composed of native individuals (79%), fishermen, or people engaged in subsistence agriculture (41%) with a malaria prevalence of 4%. The technique used here is highly specific for *C. hepatica *and the findings confirmed the presence of antibodies against this nematode in the western Brazilian Amazon, thus indicating the occurrence of infection among the sampled population. Nevertheless, in spite of its dispersion, this parasitic disease seldom seems to induce illness, since none of the patients analyzed showed symptoms of hepatic capillariasis. However, when subjects become ill [13,21-25], disease evolution is generally serious [26].

The prevalence found in the target population of this study (34.1%), independently of its reactivity (weak, median or strong), is roughly similar to the 44.2% value reported by Galvão [27] when he studied 500 low-income children in Salvador, Bahia, northeastern Brazil. Galvão [27] associated the high prevalence observed with some factors such as the low socio-economic level of the population and the possibility of rat carcasses exposing embryonated eggs in the environment, thus causing contamination [27]. However, our results show that after diluting the sera, the prevalence value dropped to 0.81%, thus suggesting that the occurrence of false-positive cases is significant when the dilution of 1/150 is used. The same phenomenon was observed by Galvão [27], who reported a drop in prevalence to 1.8% after diluting the positive sera to 1/500.

In another study with samples from 60 workers of the Vienna Zoo, Juncker-Voss [20] found a serum prevalence of 3.3% in sera diluted 1/40 using the indirect immunofluorescence technique. All serum positive workers were asymptomatic, with no hepatic enzyme alterations, and remained so until the conclusion of the study. After two months, serology was repeated and the test remained positive in only one of the patients. In order to test the accuracy of the diagnostic tool, patients' sera were further tested by ELISA and by the indirect immunofluorescence technique with antigens against *Echinococus granulosus, E. multioculares, Toxocara canis, Schistosoma mansoni, Fasciola hepatica, Trichuris trichiura and Trichinella spiralis*, showing no positive reactions and suggesting a high specificity of this test for *C. hepatica*. The authors suggest that the infections by *C. hepatica *originated from carcasses of *Mus musculus*, which were abundant in the zoo.

An experimental study with Wistar rats [19] confirmed the excellent sensitivity and specificity of the indirect immunofluorescense technique and estimated that positivity starts 15 days after infection and lasts for three months. In false-positive cases (ingestion of non- embryonated eggs) reactions are initially positive; however, dilutions equal or greater than 1/400 show a negative reaction. Moreover, infections with over 3 months of duration show negative reactions, thus suggesting a fall in antibody level as the eggs are destroyed in the hepatic tissue.

Considering these previous studies and in view of the high serum prevalence (34.1%) found at the initial dilution of 1/150 in the present study, we may conclude that this riverine population is in great contact with non-embryonated and embryonated eggs of *C. hepatica*, When positive sera were further diluted to equal or greater than 1/500, the prevalence falls to 0.81% (two patients). All patients with medium or high serology reactions, when first examined, did not present clinical symptoms nor liver alterations at the biochemical or ultrasonographic levels, thus characterizing a parasite of low pathogenicity, as many authors have proposed [13,21-25]. On the other hand, it is probable that there is a low environmental contamination with embryonated eggs, due to the high dispersion of people in this area, which could lead to infections with a low quantity of eggs and a consequent benign evolution of the disease. It must be considered that many authors have registered the contrast between the abundance of *C. hepatica *eggs in the environment and the rare occurrence of clinical cases of the disease [28-31]. These findings corroborate the low prevalence of carriers for the nematode *C. hepatica*.

Nevertheless, we must consider that since the present study focused on prevalence and since the antibodies do not remain in the blood for over 3 months [19], timely antibody detection is required. Thus, it is possible that the exposure to *C. hepatica *could have been underestimated in the sampled population.

The similarity of the serum prevalence distribution according to sex and among the three age groups indicates that the risk of exposure is equivalent among the population. This result contrasts with Galvão [27], who reported that the highest infection risk was among children, who were supposedly more exposed to eggs and rats' carcasses.

Another interesting finding is the greater serum prevalence in individuals original from the Amazon region, when compared to those original from outside the Amazon. This higher prevalence in Amazonian individuals may suggest that cultural habits (eating wild animal viscera) non-existent or less practiced by non-Amazonians is a factor to be considered in the infectivity of *C. hepatica*. However, the overall high serum prevalence found in the sampled population may be explained by the relatively few people from outside the Amazon that live in the region.

Contrary to the hypothesis formulated by Galvão [27], who proposed that people are contaminated by embryonated eggs from carcasses of urban rodents (which do not exist in the Amazon rain forest), we hypothesize that the riverine population under study can be exposed to the parasite's eggs by two non-exclusive mechanisms:

a-) ingestion of non-embryonated eggs from giblets of paca (*Agouti paca)*[2], peccaries (*Tayassu spp*.), agouti (*Dasyprocta aguti*), monkeys and other wild animals [16]. The consumption of giblet "farofa" (dish made of manioc flour browned in oil, mixed with giblets of these animals) is very common among these populations. These individuals may ingest livers contaminated with non-embryonated eggs and then act as carriers, disseminating *C. hepatica *eggs in the stool. This hypothesis is reinforced by the fact that 91.7% of the interviewed patients affirmed that they had eaten paca, agouti or peccary meat within the fifteen days prior to the interview.

b-) ingestion of environmental embryonated eggs from water or food contaminated with feces of individuals that had consumed contaminated viscera (false-positive) or with eggs from the carcasses of animals. This mechanism would be facilitated by the fact that there are no adequate methods of disposal of human excrements in the area.

These transmission routes may be investigated in the future by examining the subject's feces and investigating whether there is contamination of the soil with *C. hepatica *eggs.

Due to its particularities, our study area contrasts to other areas previously studied. Galvão [27] and Junker-Voss [20] suggested that the rodent *Mus musculus *seemed to play an important role in the transmission in their study areas. In the riverine population studied here, *M. musculus is *absent and there is no human agglomeration. Therefore, low transmission, low parasitary load and, consequently, low pathogenicity are observed.

## Conclusions

This study describes for the first time the occurrence of infection by *C. hepatica *in the Brazilian Amazon. Cultural aspects such as the diet (which includes viscera of wild animals) and the total lack of sanitation suggest in the future a somber perspective of inadequate control for the dissemination of the disease. Although *C. hepatica *showed a low level of pathogenicity and disease onset in this study, capillariaisis is severe enough to be considered a different pathology from the numerous other regional diseases that can cause fever and hepatosplenomegaly, such as malaria, trichinellosis, toxocariasis, arbovirosis, hepatitis, hepatic tumors, thyphoid fever and other helmintosis. Basic sanitation (septic tanks), together with educational orientation related to hygiene and food preparation (cooking food and avoiding the ingestion of viscera) may help to control this endemic disease. Complementary studies must be made in order to identify other wild carriers of *C. hepatica *among wild animals, in the region such as monkeys, deers, and wild rodents.

## Competing interests

The authors declare that they have no competing interests.

## Authors' contributions

LMAC: elaboration and review of the manuscript and physical examination of the patients. JSAAC: elaboration of the manuscript and laboratory tests. LJVS: elaboration of the manuscript and laboratory tests. PTC: elaboration, review of the manuscript and statistical analysis. EKT: elaboration and review of the manuscript and ultrasonographic examination. MMS: elaboration and review of the manuscript and immunofluorescence test.
